# Post-polyploidisation morphotype diversification associates with gene copy number variation

**DOI:** 10.1038/srep41845

**Published:** 2017-02-06

**Authors:** Sarah Schiessl, Bruno Huettel, Diana Kuehn, Richard Reinhardt, Rod Snowdon

**Affiliations:** 1 Department of Plant Breeding, Justus Liebig University, IFZ Research Centre for Biosystems, Land Use and Nutrition, Heinrich-Buff-Ring 26-32, 35392 Giessen, Germany; 2 Max Planck Institute for Breeding Research, Carl-von-Linné-Weg 10, 50829 Cologne, Germany

## Abstract

Genetic models for polyploid crop adaptation provide important information relevant for future breeding prospects. A well-suited model is *Brassica napus*, a recent allopolyploid closely related to *Arabidopsis thaliana*. Flowering time is a major adaptation trait determining life cycle synchronization with the environment. Here we unravel natural genetic variation in *B. napus* flowering time regulators and investigate associations with evolutionary diversification into different life cycle morphotypes. Deep sequencing of 35 flowering regulators was performed in 280 diverse *B. napus* genotypes. High sequencing depth enabled high-quality calling of single-nucleotide polymorphisms (SNPs), insertion-deletions (InDels) and copy number variants (CNVs). By combining these data with genotyping data from the Brassica 60 K Illumina® Infinium SNP array, we performed a genome-wide marker distribution analysis across the 4 ecogeographical morphotypes. Twelve haplotypes, including *Bna.FLC.A10, Bna.VIN3.A02* and the *Bna.FT* promoter on C02_random, were diagnostic for the diversification of winter and spring types. The subspecies split between oilseed/kale (*B. napus* ssp. *napus*) and swedes/rutabagas (*B. napus* ssp. *napobrassica*) was defined by 13 haplotypes, including genomic rearrangements encompassing copies of *Bna.FLC, Bna.PHYA* and *Bna.GA3ox1. De novo* variation in copies of important flowering-time genes in *B. napus* arose during allopolyploidisation, enabling sub-functionalisation that allowed different morphotypes to appropriately fine-tune their lifecycle.

Polyploid crops like wheat, potato, oats and rapeseed have been enormously successful as field crops because of their huge adaptation potential. Indeed, the fact that all flowering plants derive from ancient or recent polyploidisation events^1^,^2^ points to an enormous evolutionary advantage associated with polyploidy. On the other hand, most polyploid events do not lead to a successful establishment of a new species^3^. Understanding how polyploids achieve adaptive potential has important implication for breeding in the context of environmental change.

On the other hand, the complexity of polyploid genomes has considerably restricted large-scale genetic studies of polyploid species^4^,^5^,^6^, so broad conclusions are often drawn based on diploid model plants like *Arabidopsis thaliana*. The polyploid crop most closely related to *A. thaliana* is rapeseed (*Brassica napus*), making it an excellent system to transfer information from the model to the crop. Despite its very recent origin and strong allopolyploidisation bottleneck^6^, rapeseed can be grown from boreal to subtropical and semi-arid areas, a result of strong differentiation into distinctly different morphotypes^7^.

The morphotype with highest seed yields is the biannual winter oilseed type^8^. The prerequisites for this lifecycle are winter hardiness for winter survival, along with vernalisation requirement to avoid pre-winter flowering^7^. In subtropical areas, cultivation of semi-winter types that can be vernalised in warmer temperatures is possible^7^. Boreal or semi-arid regions have periods of low plant survival rates, either due to strong winter freezing or extreme heat stress. In these regions, annual spring types are prominent. These are neither winter-hardy nor vernalisation-dependent, and the short growing season strongly limits yield potential. *B. napus* can also be grown as beet-like forms, known as swedes or rutabagas, which form a different subspecies (ssp. *napobrassica*)^7^. Swedes are generally of winter type, however have limited winter-hardiness and require extended vernalisation to flower (Fig. 1). No wild-types of *B. napus* are known, hence the species is assumed to have arisen in cultivation^7^, with at least one origin believed to be as recent as a few hundred years ago^9^. The different cultivated forms are bred in separate breeding pools, with introgression between morphotypes only in cases of extreme introgression benefit. However, this necessitates tedious backcrossing programs to restore the required ecogeographic adaptation characters^10^. Knowledge of the factors determining lifecycle traits like vernalisation requirement and flowering time is crucial for successful exchange of genetic material between *B. napus* gene pools^10^.

Although the mechanisms of vernalisation have been studied in depth in Arabidopsis, specific winter or spring alleles were not yet defined for *B. napus*. The predominant assumption is that the underlying genetic mechanisms are identical or very similar across crucifer species. The allopolyploid *B. napus* carries two almost intact subgenomes from the ancestors *Brassica rapa* (A subgenome donor) and *Brassica oleracea* (C subgenome donor). Both ancestral subgenomes arose from a common, hexaploid ancestor, raising the theoretical copy number of Arabidopsis gene homologs to six. Due to post-polyploidisation genome reduction, the average gene copy number is 4.4^11^, whereby considerable variation has been observed among different gene families, with copy number ranging from 1 to 12^12^. Homology-driven chromosome rearrangements during allopolyploidisation are a key driver of such variation^6^,^12^. Copy number variations (CNVs) have been found to impart large phenotypic influence in several plant species like Arabidopsis^13^, wheat^14^, potato^15^ and maize^16^, but also in domestic animals^17^ and humans^18^.

In Arabidopsis, *FLOWERING LOCUS C (FLC*) is the major repressor for the activity of the central flowering transcription factor *FLOWERING LOCUS T (FT*)^19^. This gene cannot be expressed before FLC protein levels drop^19^, however when this occurs *FT* can be activated by the photoperiod pathway via the transcription factor *CONSTANS (CO*)^20^. Downregulation of *FLC* takes place at the transcriptional level. The FLC chromatin is modified and rearranged in order to stabilize a new inactive form^21^,^22^. Different mechanisms are involved in the structural regulation of *FLC* gene activity, including both autonomous regulators and the vernalisation pathway^22^,^23^. Three different mechanisms may exist for the breakdown of vernalisation requirement: (i) alteration of *FLC* regulating factors like *FRIGIDA (FRI*); (ii) alteration of *FLC* gene sequence or activity; (iii) alteration of *FLC* binding sites or *FT* promoter sequences. Arabidopsis annuals and biannuals have been found to differ either in *FRI* or in *FLC*^24^, indicating that the Arabidopsis winter-spring split is governed by the first two levels of regulation. As a consequence, research on *B. napus* vernalisation has been heavily focused on investigating *FLC* homologs^25^,^26^,^27^. Indeed, a number of QTL studies in different mapping populations have suggested *FLC* loci as candidates for flowering time in *B. napus*, including populations without vernalisation requirement^28^,^29^,^30^,^31^,^32^. Moreover, it has been reported that a transposon insertion in the first intron of *Bna.FLC.A10* is associated with the vernalisation requirement of winter-type rapeseed^27^.

The aim of the present work was therefore the definition of morphotype-specific alleles or haplotypes that might further our understanding of vernalisation control in a complex allopolyploid, and simultaneously allow breeders to successfully select for desirable lifecycle traits. By comparing results of vernalisation experiments with data from genome-wide marker distribution analysis, targeted deep-sequencing of essential flowering time regulators and the *FT* promoter, and coverage analysis to estimate CNV, we provide novel insights that reveal the complexity of post-polyploidisation morphological diversification in an important crop species.

## Material and methods

### Plant material and phenotyping

A panel of 280 genetically diverse *B. napus* inbred lines (selfed for 5 or more generations) was grown in Giessen, Germany (50° 35′ N, 8° 40′ E) in 2012. The plant material was part of the ERANET-ASSYST *B. napus* diversity set that has been described previously^33^,^34^. Winter-type rapeseed and swede accessions were grown in autumn-sown trials, whereas spring-type and semi-winter accessions were grown in spring-sown trials. Plots were sown in a completely randomized block design with a harvest plot size of 3 × 1.25 m in a single replicate (containing around 200 plants).

In a separate experiment, a selection of 33 genotypes from the same set was grown in the greenhouse under semi-controlled conditions (20 °C). These genotypes were selected to represent spring, winter and swede material with different CNV patterns for *Bna.FLC*. Twenty seeds were sown in vermiculite, before being transplanted after one week into plates in soil, with 5 replicates per treatment. Four weeks after planting, these plants were either transferred to a climate chamber for vernalisation at 4 °C and short-day conditions for 6 weeks (mild vernalisation) or 12 weeks (strong vernalisation), or kept in the greenhouse (no vernalisation). Begin of flowering (BBCH 61) was tracked daily for every single plant.

### DNA isolation

Leaf material for genomic DNA extraction was harvested in spring 2012 from the field trial in Giessen, Germany. Pooled leaf samples were taken from at least 5 different plants per genotype, immediately shock-frozen in liquid nitrogen and kept at −20 °C until extraction. Leaf material was ground in liquid nitrogen with a mortar and pestle. DNA was extracted using a common CTAB protocol modified from Doyle and Doyle (1990) as described earlier^12^. DNA concentration was determined using a Qubit fluorometer and the Qubit dsDNA BR assay kit (Life Technologies, Darmstadt, Germany) according to the manufacturer’s protocol. DNA quantity and purity was further checked on 0.5% agarose gel (3 V/cm, 0.5xTBE, 120 min).

### Selection of target genes

As described previously^12^, a set of 29 *A. thaliana* flowering time genes was selected to cover the entire genetic network controlling flowering time, including circadian clock regulators (*CYCLING DOF FACTOR 1 (CDF1*), *EARLY FLOWERING 3 (ELF3*), *GIGANTEA (GI*) and *ZEITLUPE (ZTL*)), the input pathways for vernalisation (*EARLY FLOWERING 7 (ELF7*), *EARLY FLOWERING IN SHORT DAYS (EFS*), *FLOWERING LOCUS C (FLC*), *FRIGIDA (FRI*), *SHORT VEGTATIVE PHASE (SVP*), *SUPPRESSOR OF FRIGIDA 4 (SUF4*), *TERMINAL FLOWER 2 (TFL2*), *VERNALISATION 2 (VRN2*), *VERNALISATION INSENSITIVE 3 (VIN3*)), photoperiod sensitivity (*CONSTANS (CO*), *CRYPTOCHROME 2 (CRY2*), *PHYTOCHROME A (PHYA*), *PHYTOCHROME B (PHYB*)) and gibberellin (*GIBBERELLIN-3-OXIDASE 1 (GA3ox1*)), along with downstream signal transducers (*AGAMOUS-LIKE 24 (AGL24*), *APETALA 1 (AP1*), *CAULIFLOWER (CAL*), *FLOWERING LOCUS D (FD*), *FLOWERING LOCUS T (FT*), *FRUITFUL (FUL*), *LEAFY (LFY*), *SQUAMOSA PROMOTOR PROTEIN LIKE 3 (SPL3*), *SUPPRESSOR OF CONSTANS 1 (SOC1*), *TEMPRANILLO 1 (TEM1*), *TERMINAL FLOWER 1 (TFL1*)). On top, we also included *CIRCADIAN CLOCK ASSISTED 1 (CCA1*), *FLAGELLIN-SENSITIVE 2 (FLS2*), *GLYCIN-RICH PROTEIN 7 (GRP7*), *GLYCIN-RICH PROTEIN 8 (GRP8*), *GORDITA (GORD*) and *SENSITIVITY TO RED LIGHT REDUCED 1 (SRR1*).

A full list of gene names and putative functions is provided in Supplementary Table 1.

### Bait development

In order to perform target enrichment, complementary sequences of 120 nt length were first developed for each target region. A group of 120mer oligonucleotide sequences covering a certain target region is hereinafter referred to as a bait group for that target region, while collectively all bait groups are referred to as the bait group pool. In the present study the bait group pool for the sequence capture, developed mainly using gene sequences from *B. rapa* or *B. oleracea*^12^, was modified in order to improve specificity. Enriched regions captured in our previous study^12^ were classified into target regions and non-target regions. The bait pool was then blasted against target and non-target regions with an E-value cut-off of 10^−10^. Baits which had excessive non-target hits were manually removed. This was the case for bait groups on *FT, FUL* and *PHYA*. For some bait groups (*AP1, CO, SOC1*), too many baits (>30%) were deleted. In these cases, bait groups were created using a pre-publication draft (version 4.0) of the *B. napus* ‘Darmor-Bzh’ reference genome sequence assembly, which was kindly made available prior to public release by INRA, France, Unité de Recherche en Génomique Végétale^6^, using the Agilent Genomic Workbench program SureDesign (Agilent Inc., Santa Clara, CA, USA). These replaced the corresponding bait groups developed previously using *B. rapa* or *B. oleracea*. Bait groups were created using the ‘Bait Tiling’ tool. The parameters were set as follows: Sequencing Technology: ‘Illumina’, Sequencing Protocol: ‘Paired-End long Read (75 bp+)’, ‘Use Optimized Parameters (Bait length 120, Tiling Frequency 1x)’, Avoid Overlap: ‘20’, ‘User defined genome’, ‘Avoid Standard Repeat Masked Regions’. Baits for genes on the minus-strand were developed in sense, while baits on the plus-strand were developed in antisense.

In total, 63 bait groups were created for *B. rapa* copies of the target genes, 71 bait groups for *B. oleracea* copies and 24 bait groups for *B. napus* copies.

### Sequence capture and sequencing

Custom bait production was carried out by Agilent Technologies (Agilent Inc., Santa Clara, CA, USA) using the output oligonucleotide sequences from eArrayXD. Sequence capture was performed using the SureSelectXT 1 kb–499 kb Custom Kit (Agilent Inc., Santa Clara, CA, USA) according to the manufacturer’s instructions. The resulting TruSeq DNA library (Illumina Inc., San Diego, CA, USA) was sequenced on an Illumina HiSeq 2500 sequencer at the Max Planck Institute for Breeding Research (Cologne, Germany) in 100 bp single-read mode.

### Sequence data analysis

Quality control of the raw sequencing data was performed using FASTQC. Reads were mapped onto version 4.1 of the *B. napus* ‘Darmor-Bzh’ reference genome sequence assembly^6^. Mapping was performed using the SOAPaligner algorithm^35^, with default settings and the option r = 0 to extract uniquely aligned reads. Removal of duplicates, sorting and indexing was carried out with *samtools* version 0.1.19^36^. Alignments were visualised using the IGV browser version 2.3.12^37^. Enriched regions and coverage differences were calculated using the *bedtools* software with multiBamCov^38^. Calling of single nucleotide polymorphisms (SNPs) was performed with the algorithm mpileup in the *samtools* toolkit. SNP and InDel annotation was performed using CooVar^39^. Target regions were defined using the gene annotation list from the *B. napus* ‘Darmor-bzh’ v4.1 reference genome^6^ and BLAST position results of the bait pool (E-value cut-off 10^−100^) on the mapping reference, and used to calculate the fraction of target covered. For InDel calling, a separate mapping using Bowtie2^40^ was performed, as described previously^41^. Removal of duplicates, sorting and indexing was carried out with samtools version 0.1.19. An initial InDel calling was performed using samtools mpileup, and realignment of reads around InDels was performed using GATK RealignerTargetCreator, version 3.1.1^42^. A final InDel calling was then performed as described above. InDels were filtered for a minimum mapping quality of 30 and a read depth of 10 or more using vcftools^43^.

Read coverage for each captured region was normalised as follows: normalised coverage = (number of reads per region*total length of genome)/(total number of aligned reads per genotype*average read length). Copy number variation (CNV) in a given target region was assumed if the ratio of normalised coverage(genotype)/normalised coverage(all genotypes) was smaller than 0.5 or higher than 1.5, respectively.

Sequencing data for 3 genotypes from a former experiment (Silona, Campino, Magres Pajberg)^12^ were analysed separately with the same pipeline to allow inclusion in the marker distribution analysis.

### SNP genotyping and pre-processing

The 283 accessions were genotyped using the Brassica 60 K Illumina® Infinium SNP array by TraitGenetics GmbH (Gatersleben, Germany). We used the SNP positions as published in^44^. Heterozygous calls were treated as missing values. Moreover, we used the deep sequencing data to include all confidently called SNPs in biallelic state which lay in the analysed regions. Confidently called InDels were included by coding reference alleles as AA, insertions as CC, deletions as TT and heterozygous calls as missing values. The SNP matrix from the SNP array and the SNP and InDel data from deep sequencing were combined to one single marker file and sorted by position. The subsequent marker set contained 43733 markers. After pre-processing the marker set for non-missing marker values >0.9, minor allele frequency >0.01 and individuals (genotypes) with non-missing individual markers >0.8, we retained 33944 unique SNP markers and a population of 271 individuals for marker distribution analysis. Data pre-processing was performed with R (version 3.1.0) using the package GenABEL^45^.

### Population structure

Population structure analysis and visualization were performed in R (version 3.1.0) using the package SelectionTools (http://fb09-pg-s207.agrar.uni-giessen.de/~frisch-m/), which applies principal component analysis based on genetic distances calculated according to the euclidean modified Rodger’s distance method. The most likely number of population subclusters was determined to be 3 by plotting the within-cluster sum of squares against the possible number of clusters, ranging from 1 to 15. K-means clustering was then performed in R using SelectionTools.

### Marker distribution analysis

For every marker, we counted the allele frequency of the alternative allele in each morphotype pool. The ratio between the frequency of the allele in the winter pool (winter + swedes) and the spring pool (semi-winter + spring) was used to assign the allele as a winter or spring allele. If the ratio was <1, the alternative allele was denoted spring (s), if it was >1, the alternative allele was denoted winter (w). We then first tested if the marker would be suitable to explain a morphotype split, by comparing the observed distribution of w alleles in the winter pool (without swedes) and s alleles in the spring pool (without semi-winter) with the expected distribution (139/114), using a χ^2^ test. Only markers which did not show significant deviation from this distribution (p-value > 0.1) were considered in the next step. In the next step, we tested the distribution against random distribution between the pools, by comparing the observed distribution of w alleles in winter/s alleles in spring/s alleles in winter/w alleles in spring against the expected random distribution of 69.5/57/69.5/57. We then considered the top 0.1% of −log(p-value) as split markers. The same was done for the swede and non-swede material.

## Results

### Deep sequencing and variant calling

We defined regions as genetic regions which were covered with a mean coverage in the population of at least 10. In total, we analyzed 1184 regions, of which 637 regions were annotated as genes. Of these, 184 corresponded to the intended target genes. Two target genes copies for *VERNALISATION INSENSITIVE 3 (VIN3*) (*Bna.VIN3.A01* and *Bna.VIN3.C01*) had insufficient coverage for this analysis and were not considered. Among the non-genic regions, we found 33 regions giving a BLAST hit to the *FLOWERING LOCUS T (FT*) promoter. A further 12 regions were identified as pseudogenes of the target genes. Those regions which were assigned to one of those classes (target genes, target pseudogenes and FT promoter) were summarized as target regions. A gene group is defined as all copies of a specific gene.

**Figure 2:**
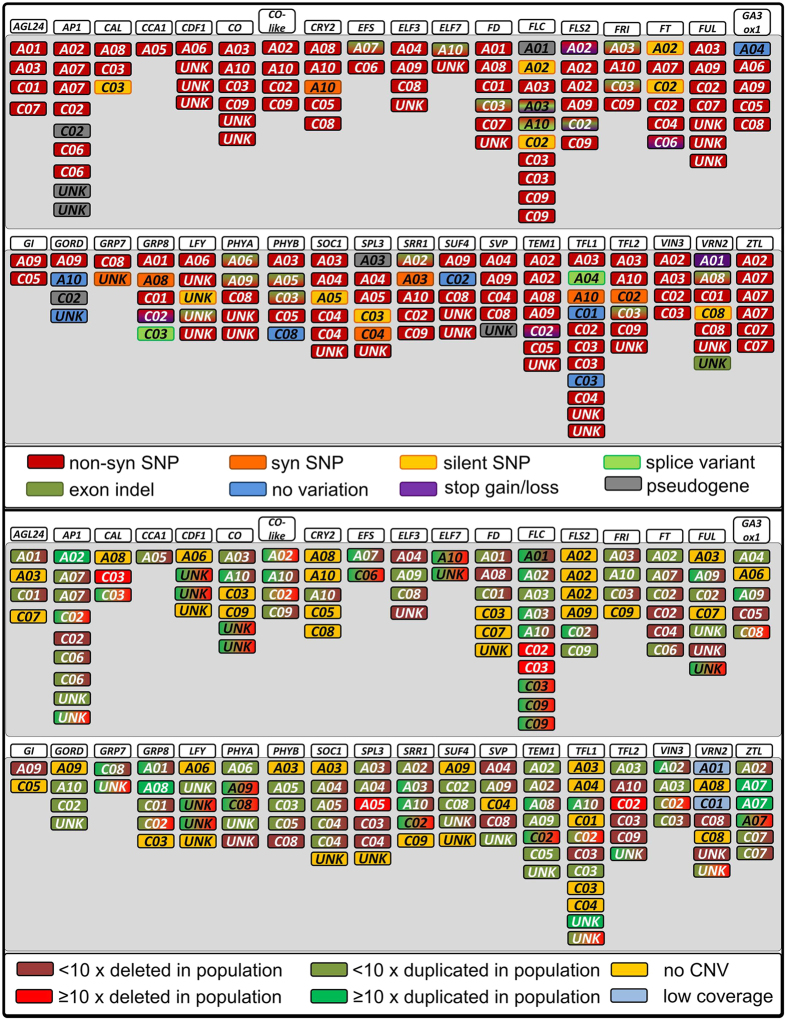
Distribution of SNPs/InDels (above) and CNV events (below) over all target gene copies. The chromosomal locations of the copies are given below the common Arabidopsis gene name (white background), with colors representing the respective type of sequence variation observed (see color code below each diagram). Upper panel: Silent SNPs are not indicated if synonymous or non-synonymous SNPs are present in the same copy, and synonymous SNPs are not indicated if non-synonymous SNPs are present in the same copy. Lower panel: Gene copies showing two different colors are deleted in some lines and duplicated in some others.

We called and annotated 13053 SNPs, of which 4806 were located in the target regions. InDel calling revealed a total of 1894 InDels, with 506 in the target regions. Only 25 InDels were frameshifts, amino acid insertions or splice variants. All gene groups showed potentially functional variation, i.e. at least one copy of the gene group carried either a non-synonymous SNP, stop codon mutation, amino acid insertion, splice variant or frameshift InDel. Altogether, only 7 copies were completely conserved, while 16 copies carried only silent or synonymous variation. Interestingly, no functional variation was observed in two copies of *Bna.FLOWERING LOCUS C (FLC*) (on chromosomes A02/C02) and two copies of *Bna.FT* (also on chromosomes A02/C02), respectively. On the other hand, other copies of *Bna.FLC* (A03, A10) and *Bna.FT* (C06) carried a surprisingly large range of variation. Among the genes with frameshift variants were copies of *Bna.FRIGIDA (FRI*), *Bna.PHYTOCHOME A (PHYA*), *Bna.EARLY FLOWERING IN SHORT DAYS (EFS*), *Bna.EARLY FLOWERING 7 (ELF7*), *Bna.PHYTOCHROM B (PHYB*), *Bna.VERNALISATION 2 (VRN2*) and *Bna.LEAFY (LFY*) (Fig. 2). We also calculated copy number variation (CNV) based on read depth. No gene group was found without CNV, and only two lines were found which did not carry any CNV among the target copies. The distribution of SNPs, InDels and CNVs is shown in Fig. 2.

### Population structure

**Figure 3:**
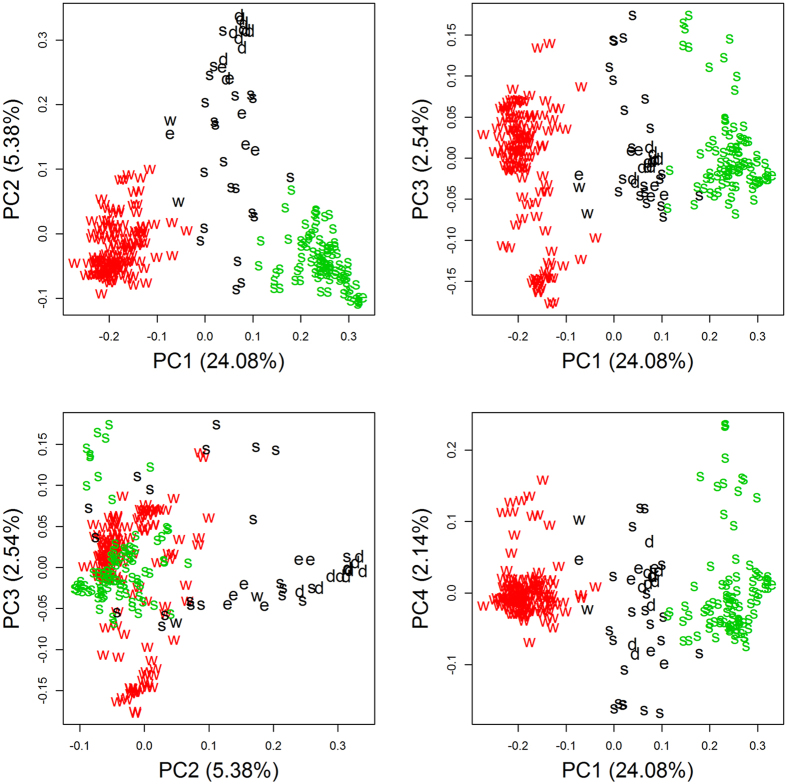
2D plots of PCA for the total population. The explained variance is given in brackets. Colors indicate the cluster. Cluster 1 is shown in red, cluster 2 in green and cluster 3 in black. Letters indicate the morphotype: w for winter, s for spring, e for semi-winter and d for swedes.

Among the analysed population of 271 accessions, we had 139 winter type accessions, 7 semi-winter type accessions, 114 spring type accessions and 11 swedes. Analyzing this population with a Principal Component Analysis (PCA) showed a strong population substructure, as the first principal component explained 24.1% of the variation, while further components explained 5.4, 2.5 and 2.1%, respectively (Fig. 3). The population falls into three main clusters: the first cluster contained 137 winter type accessions, the second one 93 spring type accessions and a semi-winter type accession, and the third and most diverse cluster contained 11 swedes, 6 semi-winter type accessions, 21 spring type accessions and 2 winter type accessions. We concluded that the winter material was genetically least diverse, while spring material was more diverse, followed by semi-winter and swede material. Overlap between winter and spring pools is minimal, while all other types show more overlap, although swedes are more distant from the winter and spring core clusters.

### Marker distribution analysis

**Figure 4:**
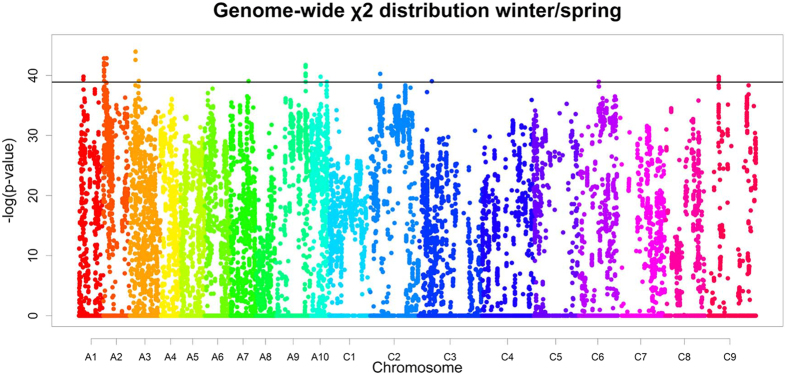
Genome-wide distribution of χ^2^ p-values tested against equal distribution in winter and spring material. The chromosomes are coloured differently. The solid lines indicate the marker cut-off threshold of 0.1%.

To analyse marker distribution on a genome-wide scale, we used SNP data from the Brassica 60 K Illumina® Infinium SNP array and combined it with data from deep sequencing (SNPs and InDels). In order to find the most indicative marker patterns for the differential flowering behaviour of winter and spring material, we analyzed the differential marker pattern between the different morphotypes using the χ^2^ test. We first defined “winter” and “spring” alleles by allele frequencies in the different morphotypes and assessed their distribution in both pools. First, we excluded all markers with non-suitable allele frequencies. We regard all markers as non-suitable if their minor allele frequency was too low to explain a population split. This was tested in a foregoing χ^2^ test (see Methods). The remaining markers were tested against random distribution in the respective morphotypes. The same was done for swedes against non-swede accessions. Choosing a cut-off which considers the top 0.1% of markers (-log[p-value] = 38.9 for winter/spring and 55.8 for swedes), we detected 12 regions on chromosomes A01, A02, A03, A07, A09, A10, C03, C06 and C09 for the winter-spring split (Fig. 4), and 13 regions on chromosomes A03, A04, A06, A09, C01, C08 and C09 for the swede split.

### Analysis of split regions

**Figure 5:**
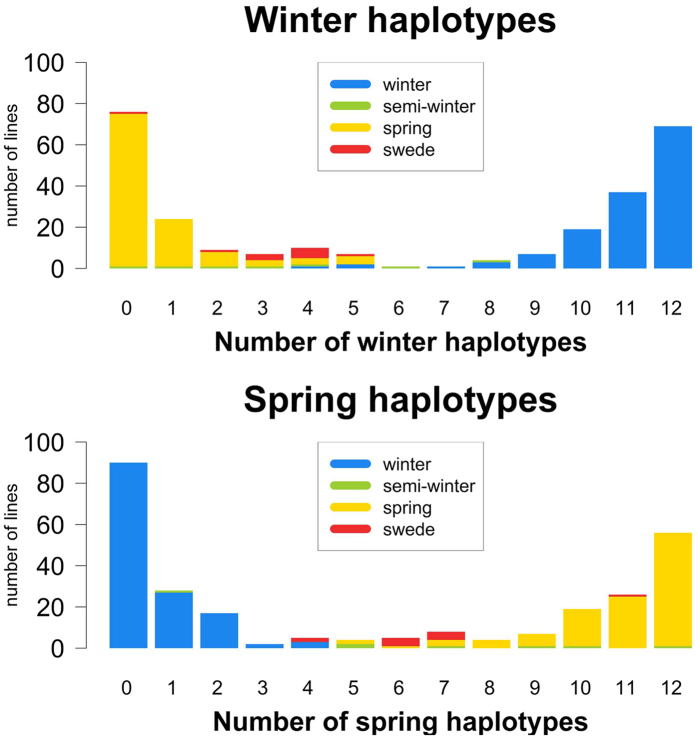
Distribution of clear winter haplotypes (above) and clear spring haplotypes (below) in the total population for all identified split regions. Mixed haplotypes were not counted. The distribution on morphotypes is colour-coded.

**Figure 6:**
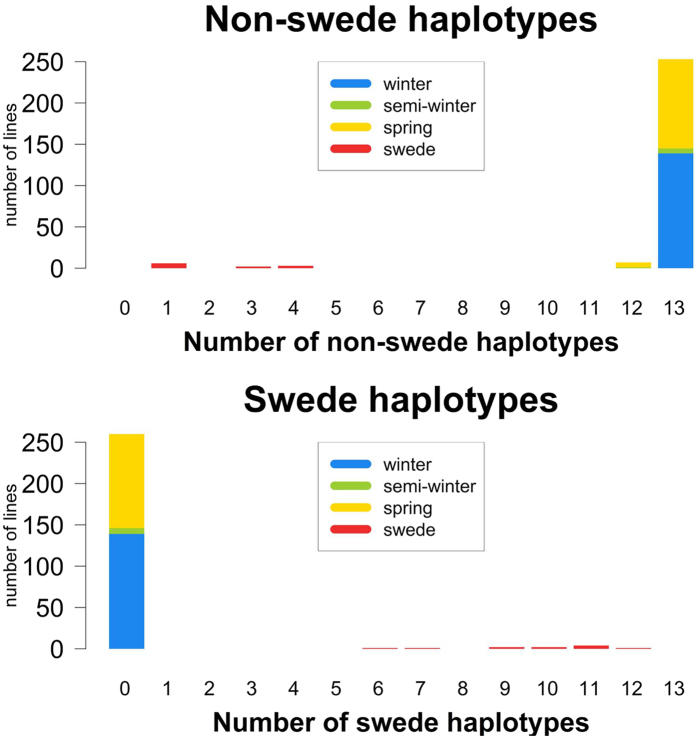
Distribution of clear non-swede haplotypes (above) and clear swede haplotypes (below) in the total population for all 13 identified split regions. Mixed haplotypes were not counted. The distribution on morphotypes is colour-coded.

We subsequently counted how many of the 12 winter-spring split regions have a clear winter or spring pattern in each genotype, i.e. the number of cases where every split marker in the haplotype corresponded to the winter or spring state (Table 1). The distribution of lines carrying clear winter and spring haplotypes is shown in Fig. 5. Mixed haplotypes were excluded here, as they account for less than 5% of the haplotypes. From this distribution, we concluded that characterizing these regions for their haplotype pattern is sufficient to distinguish winter from spring morphotypes, but not to distinguish semi-winter or swede morphotypes. The same analysis on 13 split regions identified for the swede vs. non-swede split revealed a more explicit distribution (Table and Fig. 6). Genotyping these loci is therefore sufficient to distinguish swede morphotypes from non-swedes.

In order to exclude candidates for the respective morphotype split, we specifically looked at the variant distributions from deep sequencing in our marker set. Because these derived from sequence data, a poorly fitting distribution excludes the sequence from being a major cause for this morphotype, as sequencing covers the total variation of a gene. This is not the case for data from the SNP array, as even genic SNPs are not always completely predictive for their neighbor SNP. For the winter-spring split, only 6 sequenced regions with a distribution comparable to the detected split markers could be found, among them *Bna.VIN3.A02, Bna.FLC.A10* and the *Bna.FT* promoter on the non-assembled scaffolds of C02_random (Table 1). With the exception of *Bna.FLC.A10* (R10P mutation), all those SNPs are either synonymous or located in an intron. For the non-swede vs. swede split, we found 17 sequenced regions carrying variants with an acceptable distribution, for example three copies of *Bna.FLC*, two copies of *Bna.CO* and a further copy of *Bna.FLOWERING LOCUS D (FD*) (Table 2).

**Figure 7:**
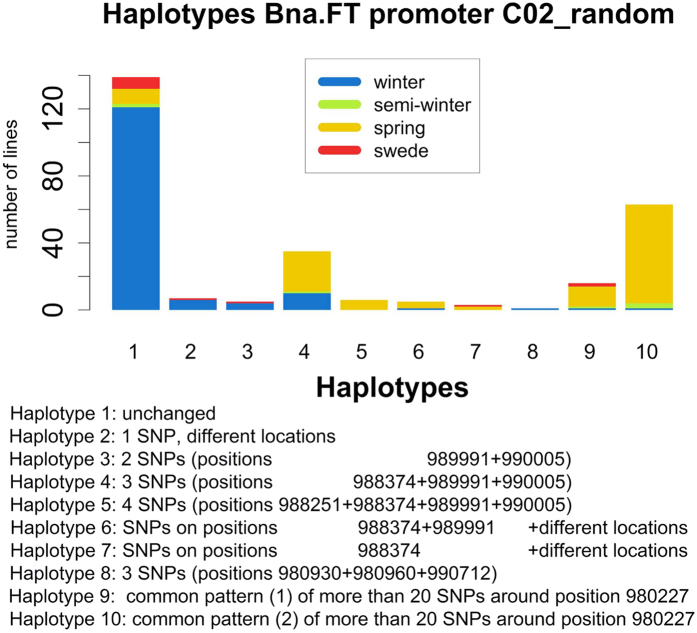
Haplotype distribution for the Bna.FT promoter on C02_random. The distribution on morphotypes is colour-coded. The haplotype patterns are provided in the text.

Upstream of the gene *Bna.FT* on C02_random we found two regions, spanning 4622 and 4904 bp, respectively, which retrieved BLAST hits to the A02 or C02 copies of the *Bna.FT* promoter listed by NCBI. We therefore identified these sequences as the promoter of *Bna.FT* on C02_random. Both sequences contain a CArG box core motif, whereby the first sequence also contains 3 additional FLC binding sites known from *A. thaliana*^46^ and the second sequence contains 2 such FLC binding sites. No SNP is located in those motifs. We found that most (143 of 145) winter types are unchanged in both sequences or carry only minor changes, whereas most (71 of 116) of the spring population carried one of two distinctive haplotype patterns involving a SNP at position C02_random:980227 (Fig. 7). These patterns were shared by only two putative winter-type accessions, one of which is an exotic accession that may not need vernalisation, whereas the other is an accession which the vernalisation experiments revealed to have vernalisation-independent flowering (see below).

We furthermore compared the numbers of deletion and duplication events in the different morphotype pools. For the winter-spring split, we found no specific pattern for the total population. In contrast, we found several patterns of deletions and duplications which were almost exclusive to swedes, concerning split regions on A08, A09, A10, C08 and C09 (Table 3). The regions on A09/C08 (containing copies of *Bna.PHYA* and *Bna.GIBBERELLIN 3 OXIDASE 1 (GA3ox1*) and A10/C09 (containing copies of *Bna.FLC*) are homeologous to each other. Some of these regions, particularly those on C08, appear to involve larger homoeologous exchanges that probably affect not only the detected genes.

For the winter-spring split, we found 234 genes with flowering-related gene ontology terms within 1 Mb of one of the diagnostic split markers. Examples are shown in Table 4. In the 13 regions showing a split between swedes and non-swedes, we found 260 candidate genes within 1 Mb. In this analysis, several split markers lay directly in candidate genes covered by deep sequencing, for example *Bna.VIN3, Bna.PHYA* (2 copies), *Bna.TEMPRANILLO1 (TEM1*), *Bna.ELF7, Bna.CONSTANS (CO*), *Bna.CO-like* or *Bna.CINNAMOYL COA REDUCTASE 1 (CCR1*) (Table 5). Some of those markers were non-synonymous SNPs (in *Bna.CCR1, Bna.TEM1, Bna.CO, Bna.CO-like*), whereas others were either synonymous or located in introns or untranslated regions (UTRs).

### Vernalisation trials

**Figure 8:**
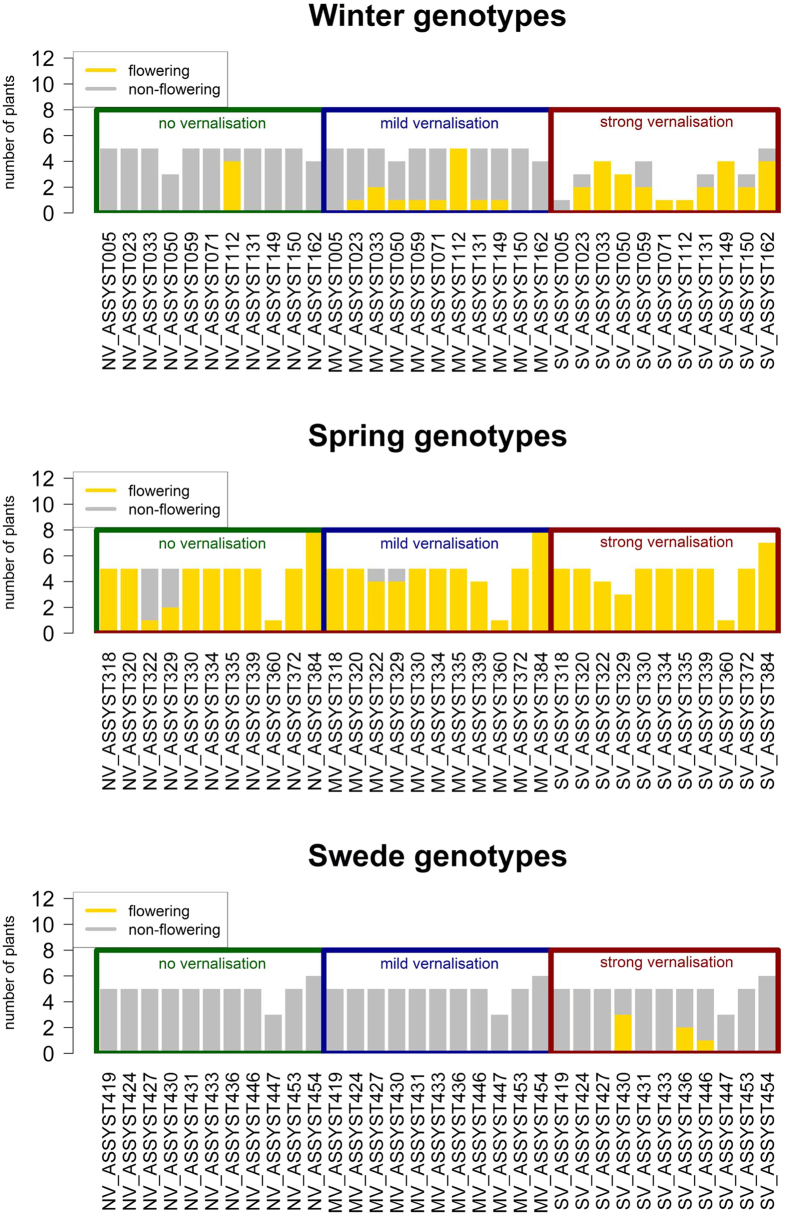
Distribution of flowering plants in the vernalisation trial. The height of the bars represents the number of replications which could be phenotyped. Yellow indicates flowering plants, grey indicates non-flowering plants. The different treatments are framed in different colors, with green indicating no vernalisation, blue mild vernalisation (6 weeks) and red strong vernalisation (12 weeks).

In order to test if the swede-specific pattern of *Bna.FLC* deletions and duplications would affect vernalisation dependency, we conducted a vernalisation trial with a reduced set of lines (11 lines each from the winter, spring and swede panels). These were selected to represent either lines without CNV in *Bna.FLC* (as a control), or lines which have alternative patterns of deletion and duplication in *Bna.FLC*. The plants were subjected to either 6 or 12 weeks of vernalisation, or were not vernalised. We then scored the time until opening of the first flower. We found that all spring lines and one winter line were vernalisation independent. The vernalisation-independent winter line was one of the two genotypes carrying a strongly divergent *Bna.FT* promoter on C02_random. All swedes and three winter lines were found to be strongly vernalisation dependent, meaning that no plants flowered after mild vernalisation. At the end of the experiment, one winter line and 8 swede lines did not flower at all, meaning that 12 weeks of vernalisation were not sufficient to induce flowering (Fig. 8).

**Figure 9:**
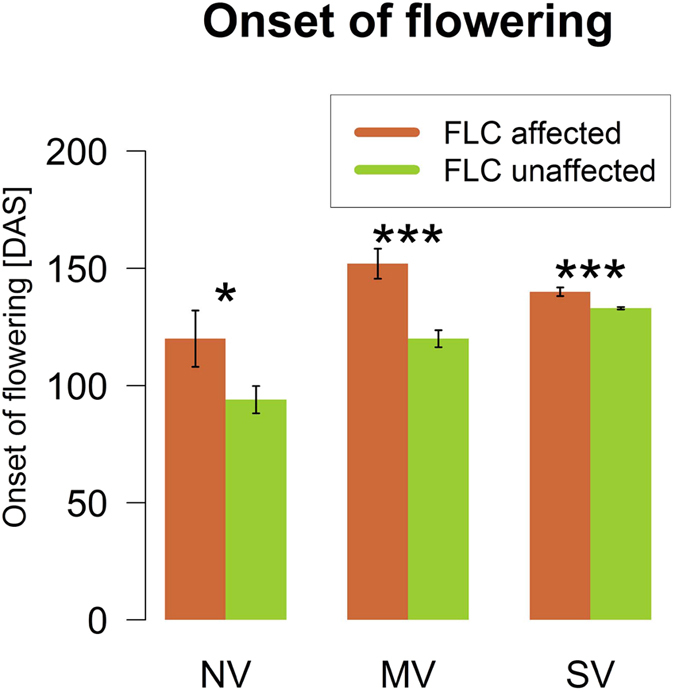
Barplots of flowering time recorded in days after sowing (DAS) for FLC-affected and non-affected plants among the spring genotypes in the vernalisation trial. Whiskers show standard errors. The asterisks denote the level of significance for Student’s t-test (*p-value < 0.05, ***p-value > 0.001). NV: No vernalisation, MV: mild vernalisation (6 weeks), SV: strong vernalisation (12 weeks).

For the spring types, we found that lines with altered *Bna.FLC* patterns flower significantly later than lines without such changes (Fig. 9). All the same, spring lines carrying a swede pattern in *Bna.FLC* were not vernalisation dependent, indicating that this pattern is not sufficient to induce vernalisation.

## Discussion

Our study aimed at identifying genetic variants which are responsible for the separation of the different morphotype pools in *B. napus*. According to population structure analyses performed in this and other studies^33^,^34^,^47^, winter-type *B. napus* accessions tend to separate almost completely from other accessions, while some spring types along with semi-winter and swede material are more diverse. Here, we defined a total of 12 variant haplotypes which are diagnostic for the winter-spring split, and 13 variant haplotypes for the swede-non-swede split. Moreover, we found one winter type without vernalisation requirement that was nevertheless winter-hardy, and spring types with some degree of vernalisation responsiveness. Swedes were found to be extremely vernalisation dependent, with some variation among the accessions, presumably because swede forms have been bred to maintain their vegetative state for as long as possible. Vernalisation is a quantitative process^48^, so there is natural variation in responsive temperature range and vernalisation duration^21^,^49^,^50^,^51^. Markers for such life cycle traits are extremely important in order to introgress desirable traits between ecogeographical or morphotype gene pools, for example seed quality traits from spring to winter oilseed forms^52^ or resistance traits from swede to non-swede material^53^.

An R10P mutation in the MADS box domain of *Bna.FLC.A10* was revealed as one candidate for the winter-spring split in *B. napus*, however our data shows that this mutation is neither the only candidate nor the best one. Neither the other *Bna.FLC* homologues, nor the detected copies of *Bna.FRI*, showed an appropriate variant distribution to explain the winter-spring split. Similar results were found for natural variation in flowering time for *A. thaliana*^54^,^55^. This excludes the possibility that genetic variation within *Bna.FLC* gene sequences, besides *Bna.FLC.A10*, are causal for vernalisation requirement, thus indicating that other *Bna.FLC* copies are either not responsible for vernalisation or there is variation in *cis*-regulatory elements. As shown by the vernalisation trials, spring-type plants without CNV in *Bna.FLC* copies flower earlier, indicating that *Bna.FLC* still plays a role in modulating flowering time in the absence of vernalisation requirement. Spring genotypes with a high *Bna.FLC* copy number showed accelerated flowering under vernalisation, indicating that they established weak vernalisation responsiveness. *FLC* is known to bind many other genes in *A. thaliana*^46^, and it also regulates other developmental processes like germination^56^, hence additional copies might be assumed to underlie strong selection. The differential degree of conservation between the copies suggests sub-functionalisation, whereby conserved *Bna.FLC.A02* and *Bna.FLC.C02* presumably retain more general roles, whereas *Bna.FLC.A10* might be more specialized towards flowering regulation. Sub-functionalisation events are characteristic for the evolution of MADS box transcription factors^57^,^58^.

On the other hand, *Bna.SRR1.A02, Bna.VIN3.A02, Bna.AGL71.A03, Bna.CCR1.A09_random* and the *Bna.FT* promoter on C02_random represent further candidates for the morphotype split. *SRR1* is a clock-associated gene found to regulate *CO, FT* and *CYCLING DOF FACTOR 1 (CDF1*) in *A. thaliana*^59^. Moreover, *A. thaliana srr1* mutants have reduced levels of *FLC* and respond only weakly to vernalisation^59^. Similarly, *Bna.VIN3.A02* represents a copy of another vernalisation candidate upstream of *Bna.FLC*^54^. In Arabidopsis, *VIN3* is expressed during cold and associates to the PRC2 complex to downregulate *FLC* gene activity^22^,^60^. *AGAMOUS-LIKE 71 (AGL71*) is closely related to the flowering integrator *SUPPRESSOR OF CONSTANS 1 (SOC1*) and seems to be involved in gibberellin-dependent flowering pathways^61^. Its promoter contains a CArG box for FLC binding in *A. thaliana*^62^. *CCR1* is a biosynthetic enzyme in lignin production and leaf development, which is known to regulate the concentration of the antioxidative compound ferulic acid^63^. This could be related to cold perception, as cold is partly perceived via the redox state^64^. Although all observed candidate SNPs are synonymous or silent, they may still have strong potential consequences for *cis*-regulatory elements, methylation, small RNA regulation and chromatin structure, and associated changes in the promoter. Moreover, alternative splicing was found to occur abundantly in resynthesized *B. napus*, also for copies of *Bna.FLC*^65^. On the other hand, we also cannot fully exclude that the observed effects are caused by linkage to additional genes in the neighbourhood of the investigated flowering-time regulators.

We also found patterns on the *Bna.FT* promoter on C02_random associating with the winter-spring split. Haplotype analysis showed that only two winter lines showed a strongly varying pattern, resembling most of the spring morphotypes. One of these was an exotic line, while the other was found to be vernalisation independent. This indicates that a functional promoter sequence for this gene is necessary to build up vernalisation requirement. A change in vernalisation requirement through variation in an *FT* promoter was already found in different *Brassicas*^66^, narrow-leafed lupine^67^ and litchi^68^. In *A. thaliana, FLC* was shown to bind to a CArG box located in the first intron of *FT*^69^, although in cereals the binding site lies in the promoter^70^. Indeed, the *Bna.FT* copy on C02_random contains a CArG box motif. It is possible that both the promoter and the intron are responsible for *Bna.FLC* binding. All the same, in both winter and spring types it has been reported^66^ that the A02 copy in *B. napus* is constitutively expressed and the C02 copy is completely silenced, whereas the copies on A07 and C06 appear to be specifically silenced in winter morphotypes but transcribed in spring morphotypes^66^. As our *Bna.FT* copy on C02_random corresponds to the C02 copy reported in the aforementioned study^66^, we assume that either there was a problem with the RT-PCR due to allelic variation, or the regulatory mechanism is more complex. However, in an independent transcriptome study we were unable to detect the constitutive expression of *Bna.FT.A02*, nor of any other *Bna.FT* copy, in winter-type *B. napus* before vernalisation (C. Obermeier, unpublished data).

Genomic rearrangements are common in *B. napus*^6^,^41^,^71^,^72^,^73^,^74^,^75^. They are particularly predominant in the first generations after allopolyploidisation^72^, but the process is ongoing and believed to have an important role in speciation^76^. Different studies found indications for genomic rearrangements between *B. napus* morphotypes^6^,^12^,^29^. In the present study we found CNVs concerning copies of *Bna.FLC, Bna.PHYA* and *Bna.GA3ox1* to involve duplications in the A subgenome and corresponding homoeologous deletions in the C subgenome. This indicates replacement of the C-subgenome regions by the respective A-subgenome regions, a process known as homeologous non-reciprocal translocation (HNRT)^77^. A *de novo* HNRT will erase any sub-functionalisation which may have occurred prior to the rearrangement. Our data concur with the hypothesis^27^ that the *Bna.FLC.A10* copy is most specifically involved in flowering regulation. A duplication in *Bna.FLC.A10* would therefore increase vernalisation requirement. This hypothesis fits with the strong vernalisation requirement we observed in lines carrying this duplication. Differential expression of *Bna.FLC* in highly rearranged, resynthesized rapeseed was observed before^26^. All the same, this pattern can only be effective when the vernalisation system is functional, as two spring lines with the same pattern are not vernalisation dependent. One of these presumably has a defective *Bna.FT* promoter on C02_random, whereas neither of them carries the swede-specific *Bna.VIN3.A03* marker.

Other genes affected by such HNRTs are *Bna.PHYA* and *Bna.GA3ox1*. PHYA is a red/far-red perceiving photoreceptor which has a stabilising role for CO under long days^78^. This might represent a necessary co-adaptation of the photoperiodic pathway due to the strong vernalisation requirement, as later flowering means that the day length is longer at the time of flowering. This assumption is underlined by the finding that a D94G mutation in a copy of *Bna.CO-like* is a candidate for the swede split. *GA3ox1*, a biosynthetic key gene involved in GA production, is regulated by PHYB and by feedback mechanisms of downstream pathways^79^. GA also affects other developmental processes like seed germination, hypocotyl elongation and fruit set^79^,^80^. *Bna.GA3ox1* is therefore also a candidate for the swede morphotype, which is characterized by an enlarged hypocotyl and low seed-set. This might also apply to *Bna.CCR1* as a candidate for the swede split. In Arabidopsis *CCR1* is involved in lignin biosynthesis, leaf development regulation and regulation of the redox state^63^ (see above).

All swede lines share a silent mutation in *Bna.VIN3.A03*, which is not shared by any other line. Although the consequence of this mutation is unclear, *Bna.VIN3* is a strong candidate for vernalisation requirement, particularly because another copy is a candidate for the winter-spring split (see above). Copies of *VIN3* have previously been named as candidates for vernalisation requirement and flowering time in *A. thaliana* and *B. napus*^54^,^81^. In *B. oleracea*, it was found that *BoVIN3* was upregulated much faster than *A. thaliana VIN3*^82^, indicating that the expression is more sensitive to cold. Another upstream candidate for the strong vernalisation requirement is *Bna.ELF7. ELF7* is involved in chromatin remodeling of *FLC* during vernalisation^83^. A further gene variant which was not found outside the swede population lay in *Bna.TEM1. TEM1* encodes another repressor of *FT*, which competes with *CO* for the same genetic region to fulfill their function^84^. The variant is a non-synonymous T167R mutation that potentially affects binding to the UTR of *FT*. A further candidate, *Bna.FD*, possibly modulates FT protein effectiveness, as FD is a direct and essential interaction partner of FT in the shoot apex^85^.

These results represent an excellent base for further experiments to transfer morphotype features between *B. napus* genetic pools. Moreover, they also shed light on the evolution of major flowering time genes in the aftermath of allopolyploidisation, and their role in morphotype diversification and ecogeographical adaptation. We clearly demonstrate that different copies of important flowering regulators play different regulatory roles across the vernalisation and flowering pathways. The scarcity of non-synonymous mutations, along with the observed variation in the *Bna.FT* promoter, underline the importance of *cis*-regulatory mechanisms in flowering time regulation.

## Additional Information

**How to cite this article**: Schiessl, S. *et al*. Post-polyploidisation morphotype diversification associates with gene copy number variation. *Sci. Rep.* **7**, 41845; doi: 10.1038/srep41845 (2017).

**Publisher's note:** Springer Nature remains neutral with regard to jurisdictional claims in published maps and institutional affiliations.

## Supplementary Material

Supplementary Table S1

## Figures and Tables

**Figure 1:**
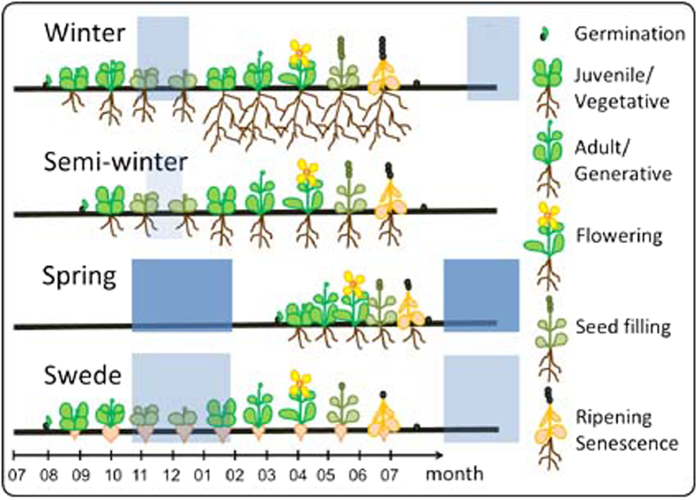
Schematic representation of the life cycles of the four different *Brassica napus* morphotypes. Periods of cold required for vernalisation in the respective morphotypes are indicated by blue boxes. Relative seed production is indicated by the number of grains.

**Table 1:** Marker distributions of SNP markers associated with the winter-spring morphotype split in *B. napus*, along with the most closely associated markers from deep sequencing for the winter-spring split.

Marker name	Chromosome	Position	winter allele		spring allele			located in gene	clear winter	clear spring	mixed	deletions
			winter pop	spring pop	winter pop	spring pop	−log(p)					
			split markers						ww	ss	ws	00
Bn-A01-p4641747	chrA01	4261277	132	13	4	101	39.3	*BnaA01g08820D*				
Bn-A01-p4641802	chrA01	4261332	132	13	4	101	39.3	*BnaA01g08820D*	136	102	12	3
**Bn-A01-p4803773**	**chrA01**	**4413051**	**128**	**9**	**6**	**105**	**39.8**					
**Bn-A02-p3207085**	**chrA02**	**695308**	**131**	**7**	**4**	**106**	**42.8**					
Bn-A02-p3208275	chrA02	700390	129	7	5	106	41.7					
Bn-A02-p3295898	chrA02	786193	130	8	5	101	39.9	*BnaA02g01700D*	129	104	12	8
Bn-A02-p3297592	chrA02	787627	128	6	6	104	40.6					
Bn-A02-p3299206	chrA02	789246	130	6	5	105	42.1	*BnaA02g01710D*				
Bn-A02-p3300731	chrA02	790766	128	6	6	105	40.9	*BnaA02g01710D*				
Bn-A02-p3302725	chrA02	792753	126	6	7	105	39.8					
Bn-A02-p3361391	chrA02	849106	126	6	8	104	39.1	*BnaA02g01860D*				
**Bn-A02-p5907701**	**chrA02**	**3096806**	**136**	**12**	**1**	**99**	**41.9**					
Bn-A02-p5917045	chrA02	3104382	137	11	2	101	42.9		148	101	2	2
Bn-A03-p6576575	chrA03	5874703	126	3	9	111	42.6	*BnaA03g12910D*	129	113	9	1
**Bn-A03-p6636780**	**chrA03**	**5928259**	**131**	**5**	**6**	**109**	**44.0**					
**Bn-A03-p9836757**	**chrA03**	**9057095**	**128**	**8**	**7**	**103**	**39.1**		136	110	na	7
**Bn-A07-p15352802**	**chrA07**	**17269795**	**126**	**7**	**9**	**106**	**39.0**	***BnaA07g22720D***	133	115	na	5
Bn-A09-p30805314	chrA09	28557636	125	4	9	107	40.2	*BnaA09g40670D*				
Bn-A09-p30805387	chrA09	28557709	125	4	9	110	41.4	*BnaA09g40670D*				
Bn-A09-p30887157	chrA09	28628531	131	11	5	103	39.9		129	101	19	4
**Bn-A09-p30909393**	**chrA09**	**28655613**	**131**	**9**	**4**	**105**	**41.7**	***BnaA09g40920D***				
Bn-A09-p30918224	chrA09	28662308	131	13	4	101	38.9					
Bn-A09-p30921980	chrA09	28664405	131	11	4	103	40.3	*BnaA09g40940D*				
**Bn-A10-p7357442**	**chrA10**	**9020292**	**128**	**7**	**9**	**106**	**39.8**	***BnaA10g10600D***				
Bn-A10-p7357555	chrA10	9020402	127	6	9	106	39.8	*BnaA10g10600D*	135	115	0	3
**Bn-scaff_17109_2-p79906**	**chrA10**	**14916811**	**121**	**1**	**17**	**111**	**38.9**	***BnaA10g21860D***	122	128	na	3
**Bn-scaff_16002_1-p1767743**	**chrC03**	**12604057**	**127**	**7**	**9**	**105**	**39.0**		134	114	na	5
**Bn-scaff_18206_3-p62755**	**chrC06**	**18959652**	**131**	**12**	**4**	**100**	**38.9**		143	104	na	6
Bn-scaff_16912_1-p190291	chrC09	12697195	129	9	8	104	39.0	*BnaC09g15770D*				
Bn-scaff_20836_1-p198809	chrC09	12804839	129	8	9	105	39.4					
Bn-scaff_20836_1-p198391	chrC09	12805246	129	8	9	105	39.4		137	112	2	2
**Bn-scaff_20836_1-p197940**	**chrC09**	**12805697**	**130**	**8**	**9**	**105**	**39.8**					
Bn-scaff_20836_1-p197387	chrC09	12806250	129	8	9	105	39.4					
Bn-scaff_20836_1-p196601	chrC09	12807036	129	8	9	104	39.0					
Regions from deep sequencing												
chrA02_3321143	chrA02	3321143	127	9	12	105	37.2	*Bna.SRR1.A02*				
chrA02_3862842	chrA02	3862842	120	3	19	111	37.1	*Bna.VIN3.A02*				
chrA03_5891342	chrA03	5891342	126	6	13	107	38.3	protein agamous-like 71				
chrA09_random_3749261	chrA09_random	3749261	126	12	13	102	34.4	*Bna.CCR1.A09_random*				
chrA10_14998726	chrA10	14998726	127	10	12	104	36.5	*Bna.FLC.A10*				
chrC02_random_990005	chrC02_random	990005	125	11	14	103	34.4	*Bna.FT.C02_random promoter*				

The table shows the marker name, chromosomal position and the number of lines carrying either a winter or a spring allele in the respective winter-type and spring-type populations. The table also gives the −log(p-value) used to determine the split markers, along with the gene ID where the marker is located. If empty, the marker is non-genic. The markers with the highest −log(p-value) in each split region are shown in bold letters. The last four columns of the table show how many clear winter or spring haplotypes were counted, along with the number of mixed haplotypes and deletions. For regions only containing one marker, mixed haplotypes do not apply (na).

**Table 2:** Marker distributions of SNP markers associated with the swede vs. non-swede morphotype split in *B. napus*, along with the most closely associated markers from deep sequencing for the swede vs. non-swede split.

Marker name	Chromosome	Position	non-swede allele		swede allele			located in gene	clear non-swede	clear swede	mixed	deletions
			non-swede pop	swede pop	non-swede pop	swede pop	−log(p)					
			split markers						nn	ss	sn	00
**chrA03_4639027**	**chrA03**	**4639027**	**260**	**0**	**0**	**11**	**57.7**	***Bna.VIN3.A03***	260	11	na	0
**chrA04_12696607**	**chrA04**	**12696607**	**0**	**8**	**260**	**3**	**55.8**		263	8	na	0
chrA06_5607262	chrA06	5607262	2	11	258	0	56.0	*Bna.CCR1.A06*	257	6	8	0
chrA06_5607744	chrA06	5607744	0	9	260	2	56.3	*Bna.CCR1.A06*				
**chrA06_5608016**	**chrA06**	**5608016**	**1**	**11**	**259**	**0**	**56.9**	***Bna.CCR1.A06***				
**chrA06_5608089**	**chrA06**	**5608089**	**1**	**11**	**259**	**0**	**56.9**	***Bna.CCR1.A06***				
chrA06_5614815	chrA06	5614815	0	8	260	3	55.8					
**chrA08_14983629**	**chrA08**	**14983629**	**260**	**3**	**0**	**8**	**55.8**	***Bna.TEM1.A08***	263	8	na	0
**chrA09_11993194**	**chrA09**	**11993194**	**0**	**8**	**260**	**3**	**55.8**	***BnaA09g19070D***	263	8	0	0
**chrA09_11993662**	**chrA09**	**11993662**	**0**	**8**	**260**	**3**	**55.8**	***BnaA09g19070D***				
**chrA09_11995810**	**chrA09**	**11995810**	**260**	**3**	**0**	**8**	**55.8**					
**Bn-A09-p21922383**	**chrA09**	**19312044**	**0**	**8**	**260**	**3**	**55.8**		263	8	na	0
chrA09_32435440	chrA09	32435440	2	11	258	0	56.0	*Bna.PHYA.A09*	258	9	4	0
chrA09_32435455	chrA09	32435455	2	11	258	0	56.0	*Bna.PHYA.A09*				
**chrA09_32437048**	**chrA09**	**32437048**	**0**	**10**	**260**	**1**	**56.9**	***Bna.PHYA.A09***				
chrA09_32441986	chrA09	32441986	260	2	0	9	56.3	*BnaA09g48430D*				
**chrA10_17106726**	**chrA10**	**17106726**	**0**	**8**	**260**	**3**	**55.8**	***Bna.ELF7.A10***	263	8	0	0
**chrA10_17106744**	**chrA10**	**17106744**	**0**	**8**	**260**	**3**	**55.8**	***Bna.ELF7.A10***				
**chrA10_17108533**	**chrA10**	**17108533**	**260**	**3**	**0**	**8**	**55.8**	***Bna.ELF7.A10***				
**chrC01_1447013**	**chrC01**	**1447013**	**0**	**8**	**260**	**3**	**55.8**		262	8	1	0
**chrC01_1447235**	**chrC01**	**1447235**	**0**	**9**	**260**	**2**	**56.3**	***Bna.FD.C01***				
**chrC01_1447273**	**chrC01**	**1447273**	**260**	**2**	**0**	**9**	**56.3**	***Bna.FD.C01***				
**chrC01_1447516**	**chrC01**	**1447516**	**0**	**9**	**260**	**2**	**56.3**	***Bna.FD.C01***				
**chrC01_1447693**	**chrC01**	**1447693**	**260**	**2**	**0**	**9**	**56.3**	***Bna.FD.C01***				
**chrC01_1447972**	**chrC01**	**1447972**	**0**	**9**	**260**	**2**	**56.3**	***Bna.FD.C01***				
**Bn-scaff_16770_1-p1357882**	**chrC08**	**24087909**	**0**	**8**	**260**	**3**	**55.8**		263	8	na	0
**chrC08_36752901**	**chrC08**	**36752901**	**260**	**1**	**0**	**10**	**56.9**	***BnaC08g42670D***	259	9	3	0
**chrC08_36752954**	**chrC08**	**36752954**	**0**	**10**	**260**	**1**	**56.9**	***BnaC08g42670D***				
chrC08_36753586	chrC08	36753586	1	10	259	1	56.1					
chrC08_36754224	chrC08	36754224	0	9	260	2	56.3	*BnaC08g42680D*				
chrC08_36755379	chrC08	36755379	1	10	259	1	56.1					
chrC08_36755562	chrC08	36755562	0	9	260	2	56.3					
**Bn-scaff_16389_1-p12505**	**chrC08**	**38113567**	**0**	**8**	**260**	**0**	**56.8**		260	8	na	3
**chrC09_43739821**	**chrC09**	**43739821**	**0**	**8**	**260**	**3**	**55.8**	***Bna.CO-li.C09***	263	8	na	0
**Regions from deep sequencing**												
chrA01_random_477115	chrA01_random	477115	5	8	255	3	51.6	mads-box protein				
chrA03_6053137	chrA03	6053137	8	9	252	2	49.6	*Bna.FRI.A03*				
chrA03_6243410	chrA03	6243410	12	8	248	2	46.2	*Bna.FLC.A03*				
chrA04_12695445	chrA04	12695445	3	8	257	3	53.3	*Bna.ELF3.A04*				
chrA05_5425314	chrA05	5425314	1	8	259	3	55.0	*Bna.SPL3.A05*				
chrA05_9211460	chrA05	9211460	4	9	256	2	52.9	ubiquitin-conjugating enzyme family protein				
chrA07_23775522	chrA07	23775522	3	8	257	3	53.3	cinnamoyl- reductase 2-2				
chrA10_1357187	chrA10	1357187	1	8	259	3	55.0	*Bna.CRY2.A10*				
chrA10_13359226	chrA10	13359226	1	9	259	2	55.4	*Bna.CO.A10*				
chrA10_14998679	chrA10	14998679	3	10	257	1	54.4	*Bna.FLC.A10*				
chrAnn_random_610372	chrAnn_random	610372	10	11	250	0	49.4	*Bna.TFL1.Ann.random*				
chrAnn_random_20504534	chrAnn_random	20504534	253	3	7	8	49.9	*Bna.VRN2.Ann.random*				
chrC03_8403949	chrC03	8403949	12	8	248	3	45.9	*Bna.FLC.C03*				
chrC03_random_5400150	chrC03_random	5400150	259	2	1	9	55.4	*Bna.FD.C03.random*				

The table shows the marker name, chromosomal position and the number of lines carrying either a non-swede or a swede allele in the respective non-swede and swede populations. The table also gives the −log(p-value) which used to determine the split markers, along with the gene ID or the name of the gene where the marker is located. If empty, the marker is non-genic. The markers with the highest −log(p-value) in each split region are shown in bold letters. The last four columns of the table show how many clear non-swede or swede haplotypes were counted, along with the number of mixed haplotypes and deletions. For regions only containing one marker, mixed haplotypes do not apply (na).

**Table 3:** Distribution of deletion and duplication events in the non-swede and swede populations.

Gene ID	Chromosome	start	stop	deletions		duplications		mean coverage	*Gene name*
				nonswede population	swede population	nonswede population	swede population		
BnaA08g15780D	chrA08	13097823	13098361	11	9	0	0	1641.5	*no annotation*
BnaA09g48410D	chrA09	32434233	32438771	31	0	10	8	1136.2	*Bna.PHYA.chrA09*
BnaA09g57140D	chrA09_random	4043861	4045464	1	0	4	8	1675.6	*Bna.GA3ox.chrA09.random*
BnaA10g22080D	chrA10	14998617	15003197	2	0	2	9	1321.5	*Bna.FLC.chrA10*
BnaC08g38580D	chrC08	34776298	34779240	9	8	1	0	1226.1	*Bna.CCR1.chrC08*
BnaC08g38810D	chrC08	34907098	34908735	4	9	0	0	1581.1	*Bna.GA3ox.chrC08*
BnaC08g42660D	chrC08	36746642	36751390	6	10	15	0	1537.7	*Bna.PHYA.chrC08*
BnaC08g42670D	chrC08	36752307	36753108	7	9	14	0	1660.2	*germin like protein*
BnaC09g46500D	chrC09	46345350	46350092	2	9	13	0	1096.9	*Bna.FLC.chrC09*
BnaC09g46540D	chrC09	46366645	46371180	3	9	11	0	1031.5	*Bna.FLC.chrC09*

The table shows the gene ID, chromosomal position and the number of lines in the respective non-swede and swede populations which carry either a deletion or duplication. The table also gives the mean coverage of the respective gene and the common gene name.

**Table 4:** Selected candidate genes for all 12 regions associated with the split between winter-type and spring-type *B. napus* accessions.

Chromosome	Marker with highest p-value	Candidate	Distance from closest split marker [kbp]
chrA01	Bn-A01-p4803773	dna topoisomerase 2	502.7
		*pseudo-response regulator 2*	*57.0*
		agamous-like protein 1	386.3
		probable lysine-specific demethylase jmj14-like	877.5
		small rna 2 -o-methyltransferase	990.8
chrA02	Bn-A02-p3207085	flowering locus c	557.1
		transcription factor hy5	342.1
		embryonic flower 1	281.6
		nuclear transcription factor y subunit a-1	127.5
		*flowering time control protein fy*	*8.1*
		zinc finger protein knuckles	44.4
		dna (cytosine-5)-methyltransferase drm2	121.7
chrA02	Bn-A02-p5917045	histone deacetylase	403.8
		auxin response factor 4	81.4
		e3 sumo-protein ligase siz1	53.5
		*ap2-like ethylene-responsive transcription factor toe2*	*1.9*
		pseudo-response regulator 3	8.2
		sensitivity to red light reduced protein	215.9
		multicopy suppressor of ira1	580.9
		vernalization insensitive 3	757.8
chrA03	Bn-A03-p6636780	protein agamous-like 71	19.3
		protein agamous-like 42	22.4
		*protein phosphatase*	8.2
		polycomb group protein embryonic flower 2	70.5
		frigida	124.9
		flowering locus c	311.8
chrA03	Bn-A03-p9836757	histone h2a	539.8
		chromatin structure-remodeling complex protein syd	445.8
		protein early flowering 4-like	164.3
		*btb poz domain-containing protein*	48.3
chrA07	Bn-A07-p15352802	*two-component response regulator arr5*	*352.9*
		sin3 histone deacetylase complex	654.6
		protein argonaute 7-like	894.1
		floral homeotic protein apetala 1	964.5
chrA09	Bn-A09-p30909393	*protein early flowering 3-like*	17.9
		e3 ubiquitin-protein ligase orthrus 2	67.9
		dna methyltransferase	727.3
chrA10	Bn-A10-p7357555	topoisomerase i	651.5
		swinger	285.1
		*histone acetyltransferase type b catalytic subunit-like*	187.2
chrA10	Bn-scaff_17109_2-p79906	transcription factor hy5	261.7
		flowering-promoting factor 1-like	99.2
		*flowering locus c*	81.8
chrC03	Bn-scaff_16002_1-p1767743	*btb poz domain-containing protein*	67.5
		*two-component response regulator arr16*	*12.8*
		phy rapidly regulated 1	743.0
chrC06	Bn-scaff_18206_3-p62755	histone z	939.5
		*squamosa-promoter binding protein*	*213.7*
		shatterproof1	749.7
chrC09	Bn-scaff_20836_1-p197940	*set domain isoform 1*	845.4

The table lists the chromosome and the marker with the most significant deviation from the expected random distribution, together with candidate genes selected based on gene ontology and literature. The last column specifies the distance of the gene to the closest marker within the split-associated region. The candidate gene closest to the split region is shown in italics.

**Table 5:** Selected candidate genes for all defined regions associated with the split between swede and non-swede *B. napus* morphotypes.

Chromosome	Marker with highest p-value	Candidate	Distance from closest split marker [kbp]
chrA03	chrA03_4639027	*vernalization insensitive 3*	*0.0*
		histone acetyltransferase type b catalytic subunit	163.4
chrA04	chrA04_12696607	protein ovule abortion 4	45.0
		*protein early flowering 3-like*	*0.1*
		terminal flowering 1 protein 1	413.7
chrA06	chrA06_5608089	gibberellin 3-oxidase	181.8
		*cinnamoyl- reductase*	*0.0*
		transcriptional factor b3 family protein	248.7
		histone acetyltransferase hac12	263.1
		protein elf4-like 4	473.9
chrA08	chrA08_14983629	*ap2-erebp rave subfamily protein rav2*	*0.0*
		cycling dof factor 2	170.7
		cullin 3	187.3
chrA09	chrA09_11993662	cullin 4	982.5
		*della protein*	*348.6*
		agamous-like mads-box protein agl3	367.7
chrA09	Bn-A09-p21922383	mads-box protein gordita	875.9
		protein suppressor of fri 4	759.6
		*nuclear transcription factor y subunit a-7*	*151.7*
chrA09	chrA09_32437048	*phytochrome a*	*0.0*
		phytochrome interacting factor 3	11.9
		histone-lysine n-methyltransferase atx2	810.9
chrA10	chrA10_17106744	probable lysine-specific demethylase elf6-like	457.6
		protein lhy cca1-like 1	51.2
		pseudo-response regulator 7	41.0
		*protein early flowering 7*	*0.0*
chrC01	chrC01_1447516	*bzip transcription factor*	*0.0*
chrC08	Bn-scaff_16770_1-p1357882	*dek domain-containing chromatin associated protein*	*942.4*
chrC08	chrC08_36752954	*phytochrome a*	*1.5*
		phytochrome interacting factor 3	10.5
		medea	551.6
chrC08	Bn-scaff_16389_1-p12505	histone-lysine n-methyltransferase atx2	434.0
		dna helicase	405.8
		*tata-box-binding protein 2*	*107.5*
chrC09	chrC09_43739821	chromo domain-containing protein lhp1-like	985.9
		della protein	866.0
		*col1 protein*	*0.0*
		coa	5.9
		sepallata2	47.5

The table lists the chromosome and the marker with the most significant deviation from the expected random distribution, together with candidate genes selected based on gene ontology and literature. The last column specifies the distance of the gene to the closest marker within the split-associated region. A value of “0.0” indicates that the marker lies within the gene. The candidate gene closest to the split region is shown in italics.

## References

[b1] Adams K. L. & Wendel J. F. Polyploidy and genome evolution in plants. Curr Opin Plant Bio 8, 135–141 (2005). 15752992 10.1016/j.pbi.2005.01.001

[b2] Soltis P. S. & Soltis D. E. The role of hybridization in plant speciation. Annu. Rev. Plant Biol. 60, 561–588 (2009). 19575590 10.1146/annurev.arplant.043008.092039

[b3] Arrigo N. & Barker M. S. Rarely successful polyploids and their legacy in plant genomes. Curr Opin Plant Bio 15, 140–146 (2012). 22480430 10.1016/j.pbi.2012.03.010

[b4] Brenchley R. . Analysis of the bread wheat genome using whole-genome shotgun sequencing. Nature 491, 705–710 (2012). 23192148 10.1038/nature11650 PMC3510651

[b5] The Potato Genome Sequencing Consortium. Genome sequence and analysis of the tuber crop potato. Nature 475, 189–195 (2011). 21743474 10.1038/nature10158

[b6] Chalhoub B. . Early allopolyploid evolution in the post-Neolithic Brassica napus oilseed genome. Science 345, 950–953 (2014). 25146293 10.1126/science.1253435

[b7] Edwards Dave, Batley Jacqueline, Parkin, Isobel & Chittaranjan Kole (ed.) Genetics, Genomics and Breeding of Oilseed Brassicas (CRC Press, 2011).

[b8] Bundessortenamt. Beschreibende Sortenliste 2016. Getreide, Mais Öl- und Faserpflanzen Leguminosen Rüben Zwischenfrüchte (2016).

[b9] Allender C. J. & King G. J. Origins of the amphiploid species Brassica napus L. investigated by chloroplast and nuclear molecular markers. BMC Plant Biol 10, 54 (2010). 20350303 10.1186/1471-2229-10-54 PMC2923528

[b10] Sharpe A. G. & Lydiate D. J. Mapping the mosaic of ancestral genotypes in a cultivar of oilseed rape (*Brassica napus*) selected via pedigree breeding. Genome 46 (2003). 10.1139/g03-031 12834063

[b11] Parkin I. A. P. Segmental Structure of the Brassica napus Genome Based on Comparative Analysis With Arabidopsis thaliana. Genetics 171, 765–781 (2005). 16020789 10.1534/genetics.105.042093 PMC1456786

[b12] Schiessl S., Samans B., Hüttel B., Reinhardt R. & Snowdon R. J. Capturing sequence variation among flowering-time regulatory gene homologs in the allopolyploid crop species Brassica napus. Front. Plant Sci. 5, 404 (2014). 25202314 10.3389/fpls.2014.00404 PMC4142343

[b13] Cao J. . Whole-genome sequencing of multiple *Arabidopsis thaliana* populations. Nat Genet 43, 956–963 (2011). 21874002 10.1038/ng.911

[b14] Díaz A. . Copy number variation affecting the photoperiod-B1 and vernalization-A1 genes is associated with altered flowering time in wheat (*Triticum aestivum*). PLoS ONE 7, 1–11 (2012). 10.1371/journal.pone.0033234 PMC3310869 22457747

[b15] Iovene M., Zhang T., Lou Q., Buell C. R. & Jiang J. Copy number variation in potato - an asexually propagated autotetraploid species. Plant J 75, 80–89 (2013). 23573982 10.1111/tpj.12200

[b16] Springer N. M. . Maize inbreds exhibit high levels of copy number variation (CNV) and presence/absence variation (PAV) in genome content. PLoS Genet 5, e1000734 (2009). 19956538 10.1371/journal.pgen.1000734 PMC2780416

[b17] Clop A., Vidal O. & Amills M. Copy number variation in the genomes of domestic animals. Anim Genet 43, 503–517 (2012). 22497594 10.1111/j.1365-2052.2012.02317.x

[b18] Żmieńko A., Samelak A., Kozłowski P. & Figlerowicz M. Copy number polymorphism in plant genomes. Theor Appl Genet. 1–18 (2013). 10.1007/s00122-013-2177-7 PMC4544587 23989647

[b19] Srikanth A. & Schmid M. Regulation of flowering time: all roads lead to Rome. Cell. Mol. Life Sci. 68, 2013–2037 (2011). 21611891 10.1007/s00018-011-0673-y PMC11115107

[b20] Kardailsky I. . Activation tagging of the floral inducer FT. Science 286 (1999). 10.1126/science.286.5446.1962 10583961

[b21] Wollenberg A. C. & Amasino R. M. Natural variation in the temperature range permissive for vernalization in accessions of Arabidopsis thaliana. Plant, Cell & Env 35, 2181–2191 (2012). 10.1111/j.1365-3040.2012.02548.x 22639792

[b22] He Y. Control of the transition to flowering by chromatin modifications. Mol. Plant 2, 554–564 (2009). 19825638 10.1093/mp/ssp005

[b23] Turck F. & Coupland G. When vernalization makes sense. Science 331, 36–37 (2011). 21212342 10.1126/science.1200786

[b24] Choi K. . The FRIGIDA complex activates transcription of FLC, a strong flowering repressor in Arabidopsis, by recruiting chromatin modification factors. The Plant Cell 23, 289–303 (2011). 21282526 10.1105/tpc.110.075911 PMC3051252

[b25] Tadege M. . Control of flowering time by FLC orthologues in *Brassica napus*. Plant J. 28 (2001). 10.1046/j.1365-313x.2001.01182.x 11849594

[b26] Pires J. C. . Flowering time divergence and genomic rearrangements in resynthesized Brassica polyploids (Brassicaceae). Biol J Linn Soc 82, 675–688 (2004).

[b27] Hou J. . A Tourist-like MITE insertion in the upstream region of the BnFLC.A10 gene is associated with vernalization requirement in rapeseed (*Brassica napus* L.) (2012). 10.1186/1471-2229-12-238 PMC3562271 23241244

[b28] Udall J. A., Quijada P. A., Lambert B. & Osborn T. C. Quantitative trait analysis of seed yield and other complex traits in hybrid spring rapeseed (*Brassica napus* L.): 2. Identification of alleles from unadapted germplasm. Theor Appl Genet 113, 597–609 (2006). 16767446 10.1007/s00122-006-0324-0

[b29] Quijada P. A., Udall J. A., Lambert B. & Osborn T. C. Quantitative trait analysis of seed yield and other complex traits in hybrid spring rapeseed (Brassica napus L.): 1. Identification of genomic regions from winter germplasm. Theor Appl Genet 113, 549–561 (2006). 16767447 10.1007/s00122-006-0323-1

[b30] Nelson M. N. . Quantitative trait loci for thermal time to flowering and photoperiod responsiveness discovered in summer annual-type *Brassica napus* L. PLoS ONE 9, e102611 (2014). 25061822 10.1371/journal.pone.0102611 PMC4111298

[b31] Raman H. . Genetic and physical mapping of flowering time loci in canola (*Brassica napus* L.). Theor Appl Genet 126, 119–132 (2013). 22955939 10.1007/s00122-012-1966-8

[b32] Fletcher R. S., Mullen J. L., Heiliger A. & McKay J. K. QTL analysis of root morphology, flowering time, and yield reveals trade-offs in response to drought in *Brassica napus*. Ex Bot J (2014). 10.1093/jxb/eru423 PMC4265167 25371500

[b33] Bus A., Hecht J., Huettel B., Reinhardt R. & Stich B. High-throughput polymorphism detection and genotyping in *Brassica napus* using next-generation RAD sequencing. BMC Genomics (2012). 10.1186/1471-2164-13-281 PMC3442993 22726880

[b34] Körber N. . Seedling development in a *Brassica napus* diversity set and its relationship to agronomic performance. Theor Appl Genet 125, 1275–1287 (2012). 22782254 10.1007/s00122-012-1912-9

[b35] Li R. . SOAP2: an improved ultrafast tool for short read alignment. Bioinformatics 25, 1966–1967 (2009). 19497933 10.1093/bioinformatics/btp336

[b36] Li H. . The Sequence Alignment/Map format and SAMtools. Bioinformatics 25, 2078–2079 (2009). 19505943 10.1093/bioinformatics/btp352 PMC2723002

[b37] Robinson J. T. . Integrative genomics viewer. Nat Biotechnol 29, 24–26 (2011). 21221095 10.1038/nbt.1754 PMC3346182

[b38] Quinlan A. R. BEDTools: The Swiss-Army Tool for Genome Feature Analysis. Curr prot bioinf 47, 11.12.1–34 (2014). 10.1002/0471250953.bi1112s47 PMC4213956 25199790

[b39] Vergara I. A., Frech C. & Chen N. CooVar: Co-occurring variant analyzer. BMC Research Notes (2012). 10.1186/1756-0500-5-615 PMC3532326 23116482

[b40] Langmead B. & Salzberg S. L. Fast gapped-read alignment with Bowtie 2. Nat Meth 9, 357–359 (2012). 10.1038/nmeth.1923 PMC3322381 22388286

[b41] Schmutzer T. . Species-wide genome sequence and nucleotide polymorphisms from the model allopolyploid plant *Brassica napus*. Scientific data 2, 150072 (2015). 26647166 10.1038/sdata.2015.72 PMC4672681

[b42] McKenna A. . The genome analysis toolkit: a mapreduce framework for analyzing next-generation DNA sequencing data. Genome Res 20, 1297–1303 (2010). 20644199 10.1101/gr.107524.110 PMC2928508

[b43] Danecek P. . The variant call format and VCFtools. Bioinformatics 27, 2156–2158 (2011). 21653522 10.1093/bioinformatics/btr330 PMC3137218

[b44] Schiessl S., Iniguez-Luy F., Qian W. & Snowdon R. J. Diverse regulatory factors associate with flowering time and yield responses in winter-type *Brassica napus*. BMC Genomics 16, 737 (2015). 26419915 10.1186/s12864-015-1950-1 PMC4589123

[b45] Aulchenko Y. S., Ripke S., Isaacs A. & van Duijn C. M. GenABEL: an R library for genome-wide association analysis. Bioinformatics 23, 1294–1296 (2007). 17384015 10.1093/bioinformatics/btm108

[b46] Deng W. . FLOWERING LOCUS C (FLC) regulates development pathways throughout the life cycle of *Arabidopsis*. PNAS 108, 6680–6685 (2011). 21464308 10.1073/pnas.1103175108 PMC3081018

[b47] Bus A., Korber N., Snowdon R. J. & Stich B. Patterns of molecular variation in a species-wide germplasm set of *Brassica napus*. Theor Appl Genet 123, 1413–1423 (2011). 21847624 10.1007/s00122-011-1676-7

[b48] Song J., Irwin J. & Dean C. Remembering the prolonged cold of winter. Current Biology 23, R807–R811 (2013). 24028964 10.1016/j.cub.2013.07.027

[b49] Duncan S. . Seasonal shift in timing of vernalization as an adaptation to extreme winter. eLife (2015). 10.7554/eLife.06620 PMC4532801 26203563

[b50] Coustham V. . Quantitative modulation of polycomb silencing underlies natural variation in vernalization. Science 337, 584–587 (2012). 22798408 10.1126/science.1221881

[b51] Meyer S. E., Nelson D. L. & Carlson S. L. Ecological genetics of vernalization response in *Bromus tectorum* L. (*Poaceae*). Ann Bot 93, 653–663 (2004). 15087300 10.1093/aob/mch088 PMC4242293

[b52] Wu G., Wu Y., Xiao L., Li X. & Lu C. Zero erucic acid trait of rapeseed (*Brassica napus* L.) results from a deletion of four base pairs in the *fatty acid elongase 1* gene. Theor Appl Genet 116, 491–499 (2008). 18075728 10.1007/s00122-007-0685-z

[b53] Hasan M. J. & Rahman H. Genetics and molecular mapping of resistance to *Plasmodiophora brassicae* pathotypes 2, 3, 5, 6, and 8 in rutabaga (*Brassica napus* var. *napobrassica*). Genome 59, 805–815 (2016). 27549861 10.1139/gen-2016-0034

[b54] Dittmar E. L., Oakley C. G., Agren J. & Schemske D. W. Flowering time QTL in natural populations of *Arabidopsis thaliana* and implications for their adaptive value. Mol Ecology 23, 4291–4303 (2014). 10.1111/mec.12857 25039363

[b55] Grillo M. A., Li C., Hammond M., Wang L. & Schemske D. W. Genetic architecture of flowering time differentiation between locally adapted populations of *Arabidopsis thaliana*. New Phytol 197, 1321–1331 (2013). 23311994 10.1111/nph.12109

[b56] Chianga G. C. K., Baruaa D., Kramera E. M., Amasinob R. M. & Donohuea K. Major flowering time gene, *FLOWERING LOCUS C*, regulates seed germination in *Arabidopsis thaliana*. PNAS 106, 11661–11666 (2009). 19564609 10.1073/pnas.0901367106 PMC2710639

[b57] Becker A. The major clades of MADS-box genes and their role in the development and evolution of flowering plants. Mol Phylogen Evol 29, 464–489 (2003). 10.1016/s1055-7903(03)00207-0 14615187

[b58] Dreni L. & Kater M. M. MADS reloaded: evolution of the AGAMOUS subfamily genes. New Phytol 201, 717–732 (2014). 24164649 10.1111/nph.12555

[b59] Johansson M. & Staiger D. SRR1 is essential to repress flowering in non-inductive conditions in *Arabidopsis thaliana*. Ex Bot J 65, 5811–5822 (2014). 10.1093/jxb/eru317 PMC4203120 25129129

[b60] Wood C. C. . The *Arabidopsis thaliana* vernalization response requires a polycomb-like protein complex that also includes VERNALIZATION INSENSITIVE 3. PNAS 103, 14631–14636 (2006). 16983073 10.1073/pnas.0606385103 PMC1600011

[b61] Dorca-Fornell C. . The Arabidopsis SOC1-like genes AGL42, AGL71 and AGL72 promote flowering in the shoot apical and axillary meristems. Plant J 67, 1006–1017 (2011). 21609362 10.1111/j.1365-313X.2011.04653.x

[b62] Helliwell C. A., Wood C. C., Robertson M., James Peacock W. & Dennis E. S. The Arabidopsis FLC protein interacts directly *in vivo* with SOC1 and FT chromatin and is part of a high-molecular-weight protein complex. Plant J 46, 183–192 (2006). 16623882 10.1111/j.1365-313X.2006.02686.x

[b63] Xue J. . CCR1, an enzyme required for lignin biosynthesis in Arabidopsis, mediates cell proliferation exit for leaf development. Plant J 83, 375–387 (2015). 26058952 10.1111/tpj.12902

[b64] Kovi M. R., Ergon A. & Rognli O. A. Freezing tolerance revisited-effects of variable temperatures on gene regulation in temperate grasses and legumes. Curr Opin Plant Bio 33, 140–146 (2016). 27479037 10.1016/j.pbi.2016.07.006

[b65] Zhou R., Moshgabadi N. & Adams K. L. Extensive changes to alternative splicing patterns following allopolyploidy in natural and resynthesized polyploids. PNAS 108, 16122–16127 (2011). 21900601 10.1073/pnas.1109551108 PMC3179116

[b66] Wang J. . Promoter Variation and Transcript Divergence in Brassicaceae Lineages of FLOWERING LOCUS T. PLoS ONE 7, e47127 (2012). 23071733 10.1371/journal.pone.0047127 PMC3469537

[b67] Nelson M. N. . The loss of vernalization requirement in narrow-leafed lupin is associated with a deletion in the promoter and de-repressed expression of a *Flowering Locus T (FT*) homologue. New Phytol (2016). 10.1111/nph.14094 27418400

[b68] Ding F. . Promoter difference of *LcFT1* is a leading cause of natural variation of flowering timing in different litchi cultivars (*Litchi chinensis* Sonn.). Plant science 241, 128–137 (2015). 26706065 10.1016/j.plantsci.2015.10.004

[b69] Searle I. . The transcription factor FLC confers a flowering response to vernalization by repressing meristem competence and systemic signaling In Arabidopsis. Genes & Dev 20, 898–912 (2006). 16600915 10.1101/gad.373506 PMC1472290

[b70] Deng W. . Direct links between the vernalization response and other key traits of cereal crops. Nat Comms 6, 5882 (2015). 10.1038/ncomms6882 25562483

[b71] Clarke W. E. . A high-density SNP genotyping array for *Brassica napus* and its ancestral diploid species based on optimised selection of single-locus markers in the allotetraploid genome. Theor Appl Genet 129, 1887–1899 (2016). 27364915 10.1007/s00122-016-2746-7 PMC5025514

[b72] Gaeta R. T., Pires J. C., Iniguez-Luy F., Leon E. & Osborn T. C. Genomic Changes in Resynthesized *Brassica napus* and Their Effect on Gene Expression and Phenotype. The Plant Cell 19, 3403–3417 (2007). 18024568 10.1105/tpc.107.054346 PMC2174891

[b73] Nicolas S. D., Monod H., Eber F., Chevre A.-M. & Jenczewski E. Non-random distribution of extensive chromosome rearrangements in *Brassica napus* depends on genome organization. Plant J 70, 691–703 (2012). 22268419 10.1111/j.1365-313X.2012.04914.x

[b74] Udall J. A., Quijada P. A. & Osborn T. C. Detection of Chromosomal Rearrangements Derived From Homeologous Recombination in Four Mapping Populations of *Brassica napus* L. Genetics 169, 967–979 (2005). 15520255 10.1534/genetics.104.033209 PMC1449096

[b75] Szadkowski E. . Polyploid formation pathways have an impact on genetic rearrangements in resynthesized *Brassica napus*. New Phytol 191, 884–894 (2011). 21517871 10.1111/j.1469-8137.2011.03729.x

[b76] Faria R. & Navarro A. Chromosomal speciation revisited: rearranging theory with pieces of evidence. Trends ecol evol 25, 660–669 (2010). 20817305 10.1016/j.tree.2010.07.008

[b77] Gaeta R. T. & Pires J. C. Homoeologous recombination in allopolyploids: the polyploid ratchet. New Phytol 186, 18–28 (2010). 20002315 10.1111/j.1469-8137.2009.03089.x

[b78] Valverde F. . Photoreceptor regulation of CONSTANS protein in photoperiodic flowering. Science 303, 1003–1006 (2004). 14963328 10.1126/science.1091761

[b79] Gabriele S. . The Dof protein DAG1 mediates PIL5 activity on seed germination by negatively regulating GA biosynthetic gene AtGA3ox1. Plant J 61, 312–323 (2010). 19874540 10.1111/j.1365-313X.2009.04055.x

[b80] Gallego-Giraldo C. . Role of the gibberellin receptors GID1 during fruit-set in Arabidopsis. Plant J 79, 1020–1032 (2014). 24961590 10.1111/tpj.12603 PMC4403254

[b81] Shi J. . Unraveling the Complex Trait of Crop Yield With Quantitative Trait Loci Mapping in *Brassica napus*. Genetics 182, 851–861 (2009). 19414564 10.1534/genetics.109.101642 PMC2710164

[b82] Ridge S., Brown P. H., Hecht V., Driessen R. G. & Weller J. L. The role of BoFLC2 in cauliflower (*Brassica oleracea* var. *botrytis* L.) reproductive development. Ex Bot J 66, 125–135 (2015). 10.1093/jxb/eru408 PMC4265156 25355864

[b83] He Y., Doyle M. R. & Amasino R. M. PAF1-complex-mediated histone methylation of *FLOWERING LOCUS C* chromatin is required for the vernalization-responsive, winter-annual habit in *Arabidopsis*. Genes & Dev 18, 2774–2784 (2004). 15520273 10.1101/gad.1244504 PMC528897

[b84] Castillejo C. & Pelaz S. The Balance between CONSTANS and TEMPRANILLO Activities Determines *FT* Expression to Trigger Flowering. Current Biology 18, 1338–1343 (2008). 18718758 10.1016/j.cub.2008.07.075

[b85] Abe M. . FD, a bZIP Protein Mediating Signals from the Floral Pathway Integrator FT at the Shoot Apex. Science 309, 1052–1056 (2005). 16099979 10.1126/science.1115983

