# Supplementary material for: Post-polyploidisation morphotype diversification associates with gene copy number variation
Source: Sci Rep. 2017 Feb 6;7:41845. doi: 10.1038/srep41845 (PMC5292959; doi:10.1038/srep41845)
Supplement: Supplementary Table S1 [file srep41845-s1.docx]

Post-polyploidisation morphotype diversification associates with gene copy number variation

Sarah Schiessl^11 §^, Bruno Huettel^2^, Diana Kuehn^2^, Richard Reinhardt^2^, Rod Snowdon^1^

^1^ Department of Plant Breeding, Justus Liebig University, IFZ Research Centre for Biosystems, Land Use and Nutrition, Heinrich-Buff-Ring 26-32, 35392 Giessen, Germany

^2^ Max Planck Institute for Breeding Research, Carl-von-Linné-Weg 10

50829 Cologne, Germany

^§^ Corresponding author:

E-mail: [sarah-veronica.schiessl@agrar.uni-giessen.de](mailto:sarah-veronica.schiessl@agrar.uni-giessen.de)

Phone: 0049-641-9937445

# Supplementary information

Table S1: Gene acronyms of all sequenced genes along with their full name, *A. thaliana* locus tag and database description.

The aligned sequence data are stored at the NCBI SRA database, SRP-Study accession: SRP087610
